# Supplementary material for: Origin and diversity of Capsella bursa-pastoris from the genomic point of view
Source: BMC Biol. 2024 Mar 5;22:52. doi: 10.1186/s12915-024-01832-1 (PMC10913212; doi:10.1186/s12915-024-01832-1)
Supplement: Supplementary file 1 — Additional file 1: Fig. S1. Scheme for data acquisition for the genetic map. Fig. S2. An example of a contig fragment with a colored state of the markers. Due to the low coverage some of the markers are "noisy". Fig. S3. An example of chimeric assembly. Two adjacent markers located at a distance of ~90 kbp have 33 "recombinations" per 100 chromosomes, which is impossible and indicates independent inheritance of markers.Fig. S4. An example of correction of a local chimeric assembly. a Insertion of a foreign fragment(s) and b view of the site after correction. The green dashed lines show the signals indicating the proximity of the sites. Fig. S5. Simulation of the subgenome separation procedure. Example of the coverage by reads of the parental species of some reference contigs created from the genomes of C. orientalis and C. rubella for subgenome separation. Fig. S6. Analysis of introgression by admixture analysis in C. bursa-pastoris, for K=6. The colors of the line names correspond to the populations in Fig. 4b.Fig. S7. Fbranch matrix plotted using Dsuite f4-statistics results for different tree topologies of parental species and lineages of C. bursa-pastoris for subgenome O. a for ((((ME,EU),ASI),Co),Cgr-Outgroup) tree; b for ((((ASI,EU),ME),Co),Cgr-Outgroup) tree; c for ((((ASI,ME),EU),Co),Cgr-Outgroup) tree; d for (((ME,EU),ASI),Co-Outgroup) tree; e for (((ASI,EU),ME),Co-Outgroup) tree; f for (((ASI,ME),EU),Co-Outgroup) tree. Co – C. orientalis, Cgr – C. rubella/C. grandiflora, and ASI, ME, EU – lineages of C. bursa-pastoris. Fig. S8. Fbranch matrix plotted using Dsuite f4-stastics results for different tree topologies of parental species and lineages of C. bursa-pastoris for subgenome R. a for ((((ME,EU),ASI),Cgr),Co-Outgroup) tree; b for ((((ASI,EU),ME),Cgr),Co-Outgroup) tree; c for ((((ASI,ME),EU),Cgr),Co-Outgroup) tree; d for (((ME,EU),ASI),Cgr-Outgroup) tree; e for (((ASI,EU),ME),Cgr-Outgroup) tree; f for (((ASI,ME),EU),Cgr-Outgroup) tree. Co – C. orientalis [file 12915_2024_1832_MOESM1_ESM.pdf]

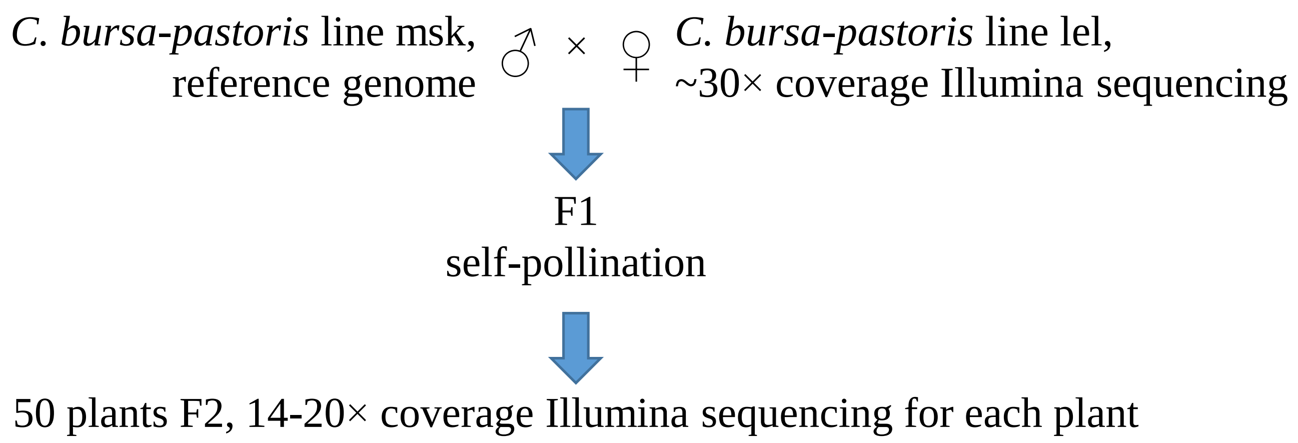

Figure S1. Scheme for data acquisition for the genetic map.

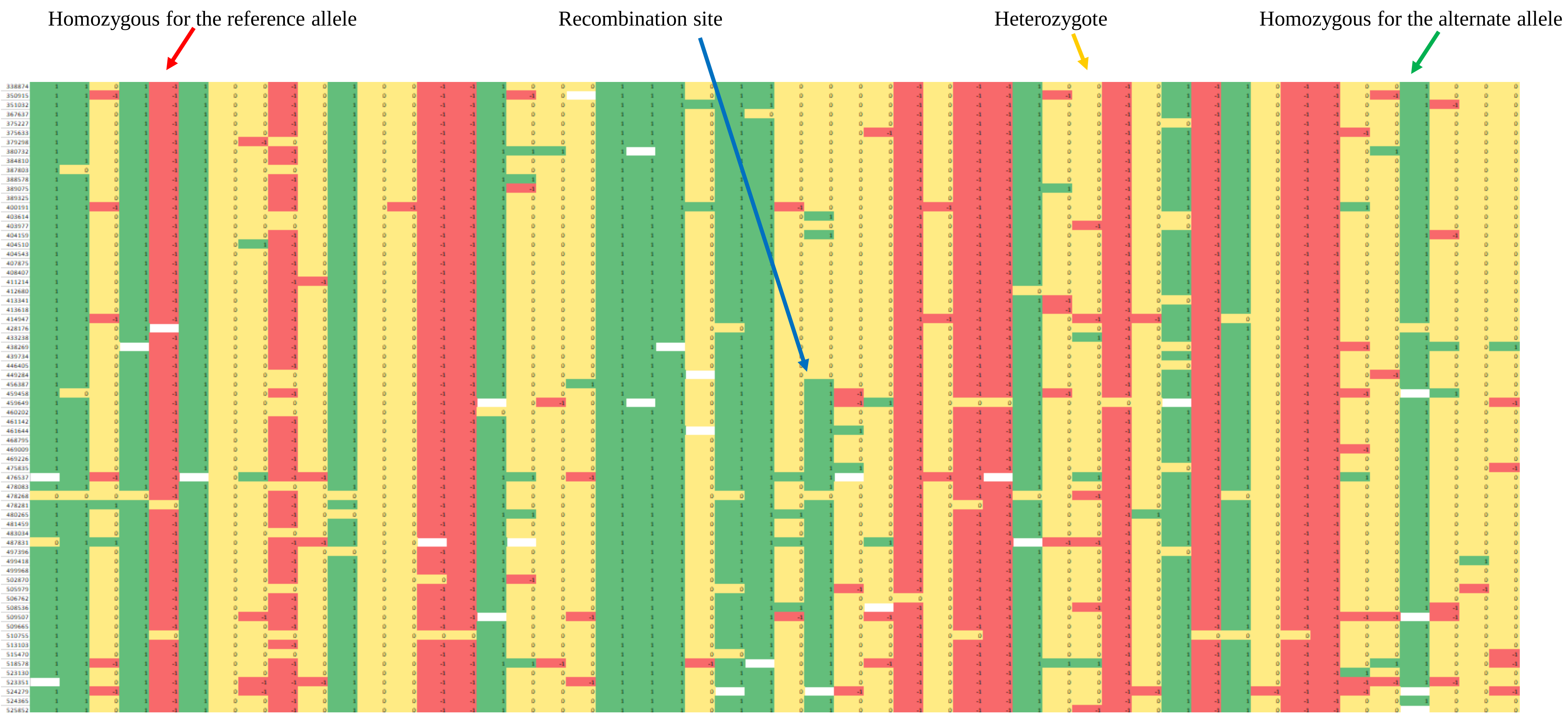

Figure S2. An example of a contig fragment with a colored state of the markers. Due to the low coverage some of the markers are "noisy".

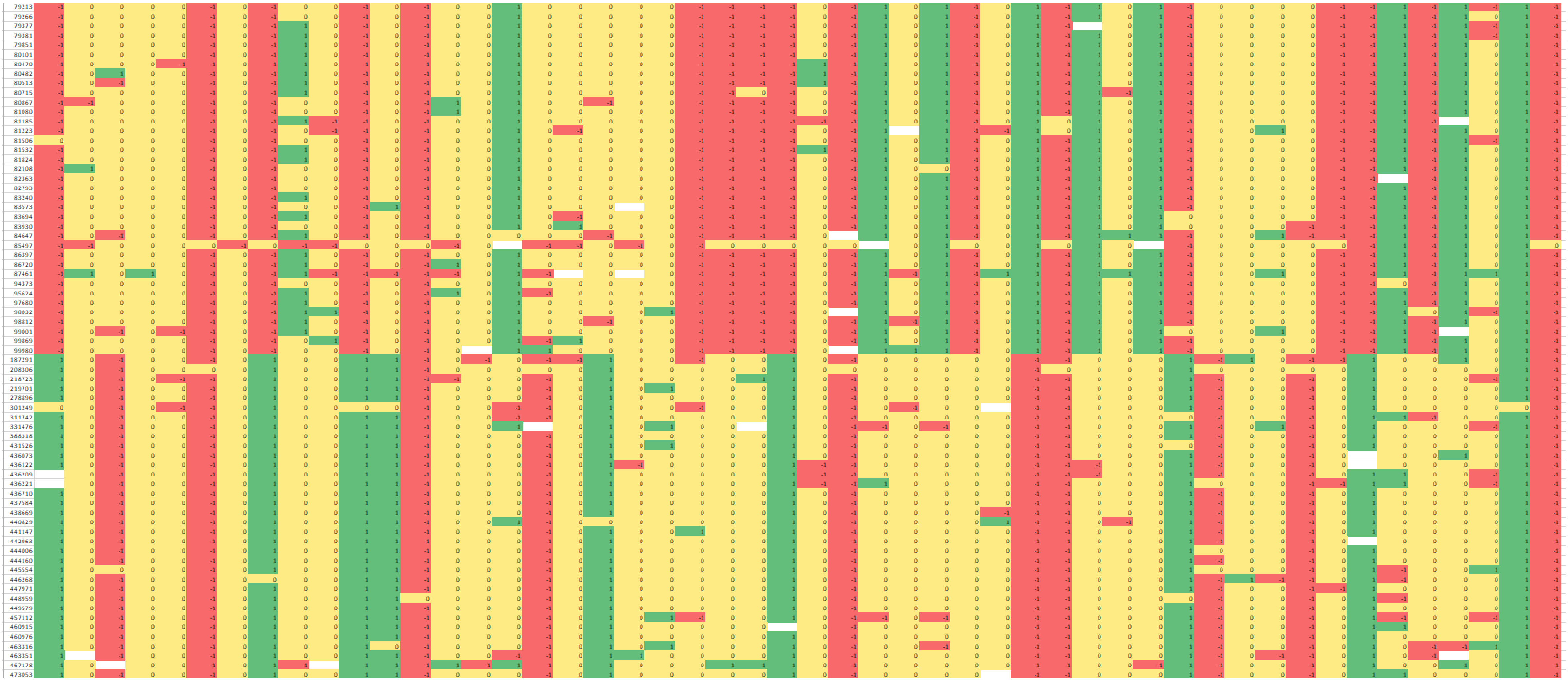

Figure S3. An example of chimeric assembly. Two adjacent markers located at a distance of ~90 kbp have 33 "recombinations" per 100 chromosomes, which is impossible and indicates independent inheritance of sites.

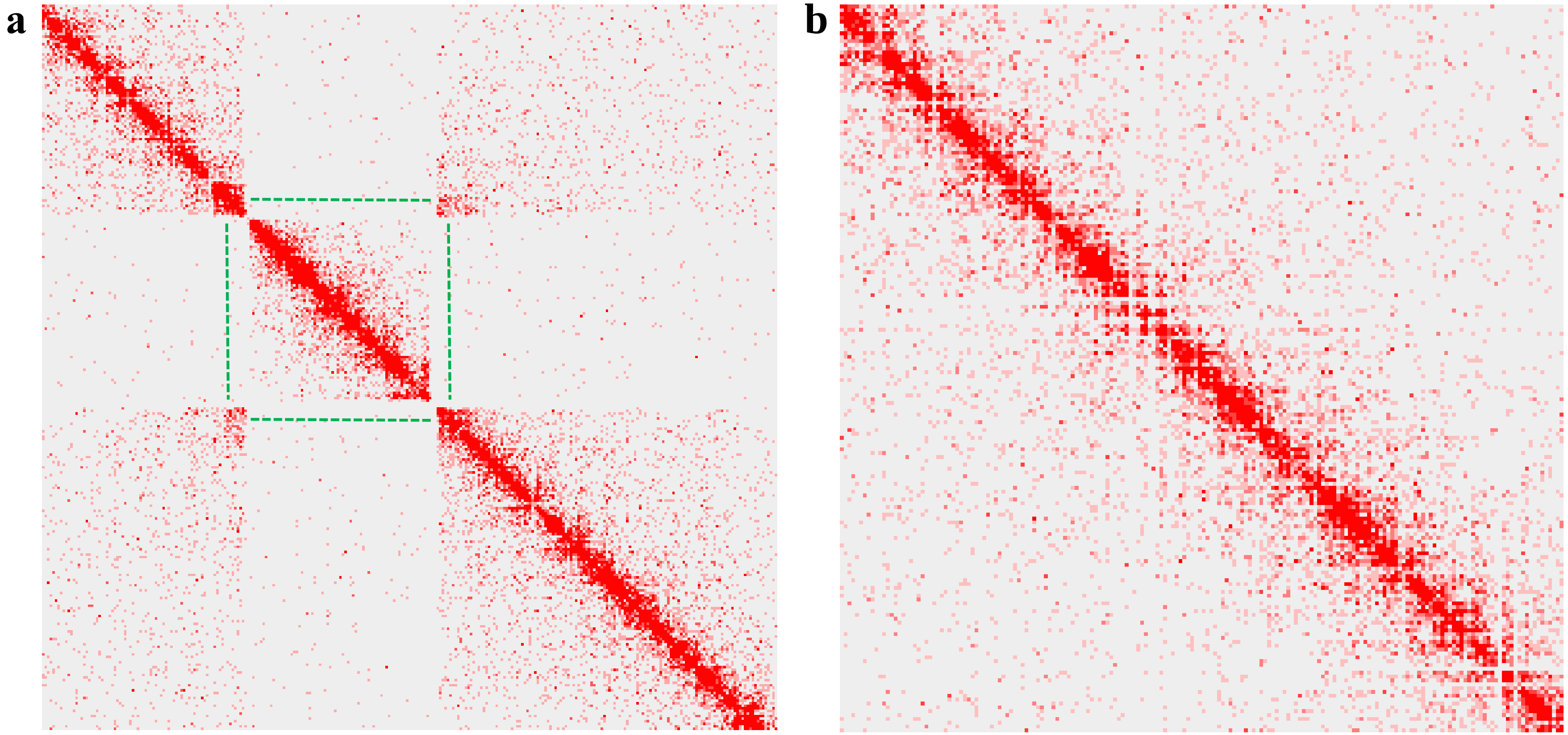

Figure S4. An example of correction of a local chimeric assembly. a Insertion of a foreign fragment(s) and b view of the site after correction. The green dashed lines show the signals indicating the proximity of the sites.

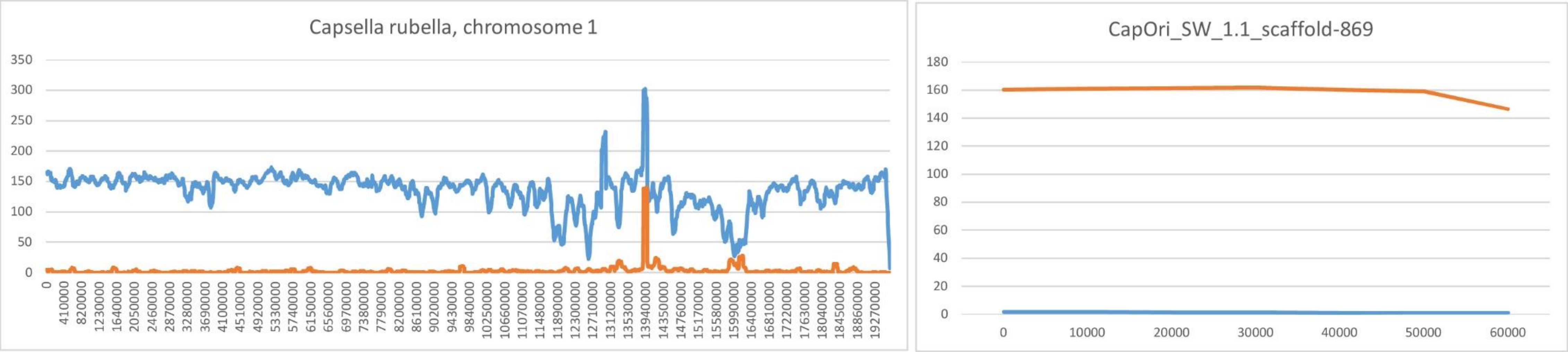

Figure S5. Simulation of the subgenome separation procedure. Example of the coverage by reads of the parental species of some reference contigs created from the genomes of *C. orientalis* and *C. rubella* for subgenome separation.

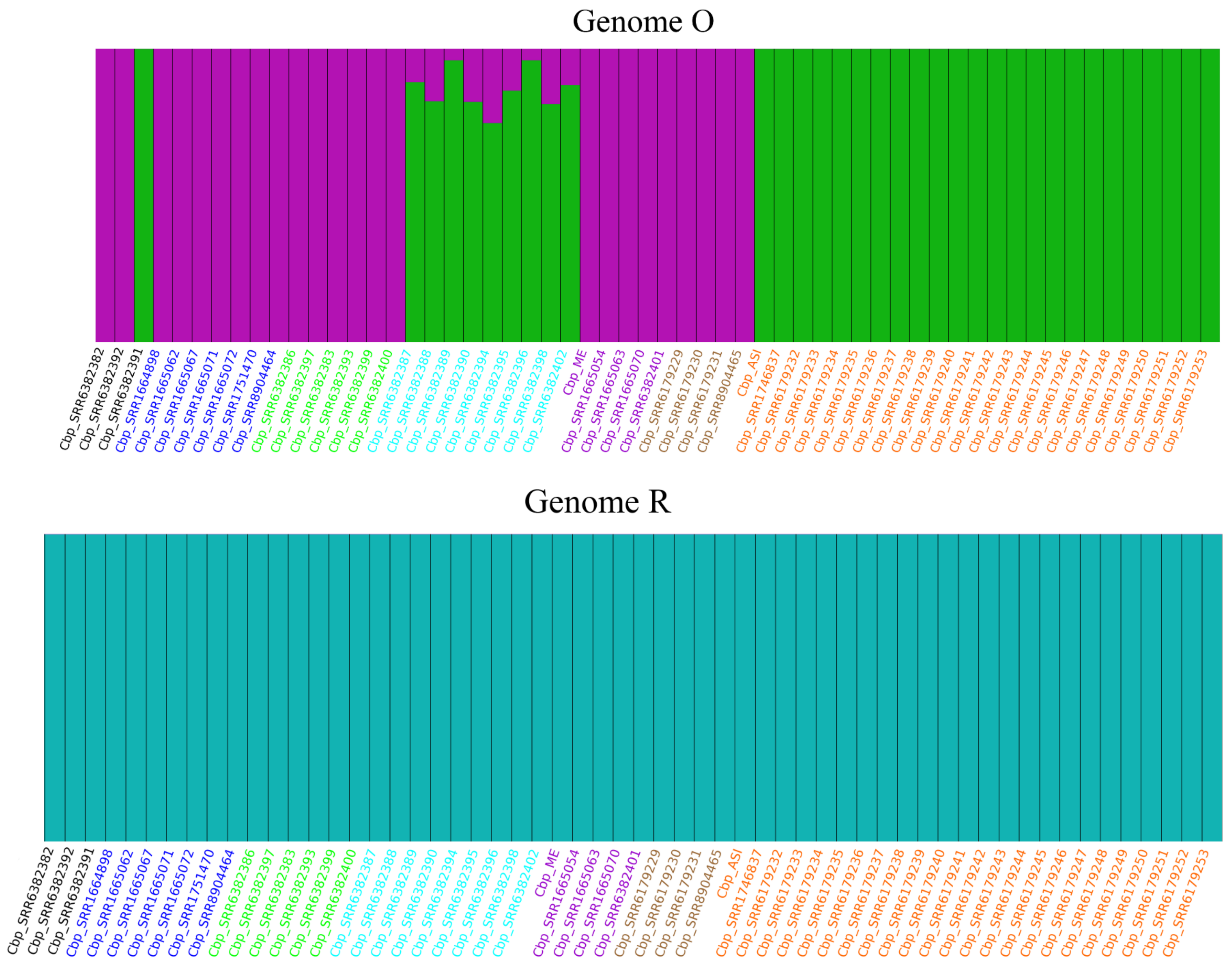

Figure S6. Analysis of introgression by admixture analysis in *C. bursa-pastoris*, for K=6. The colors of the line names correspond to the populations in Figure 4b.

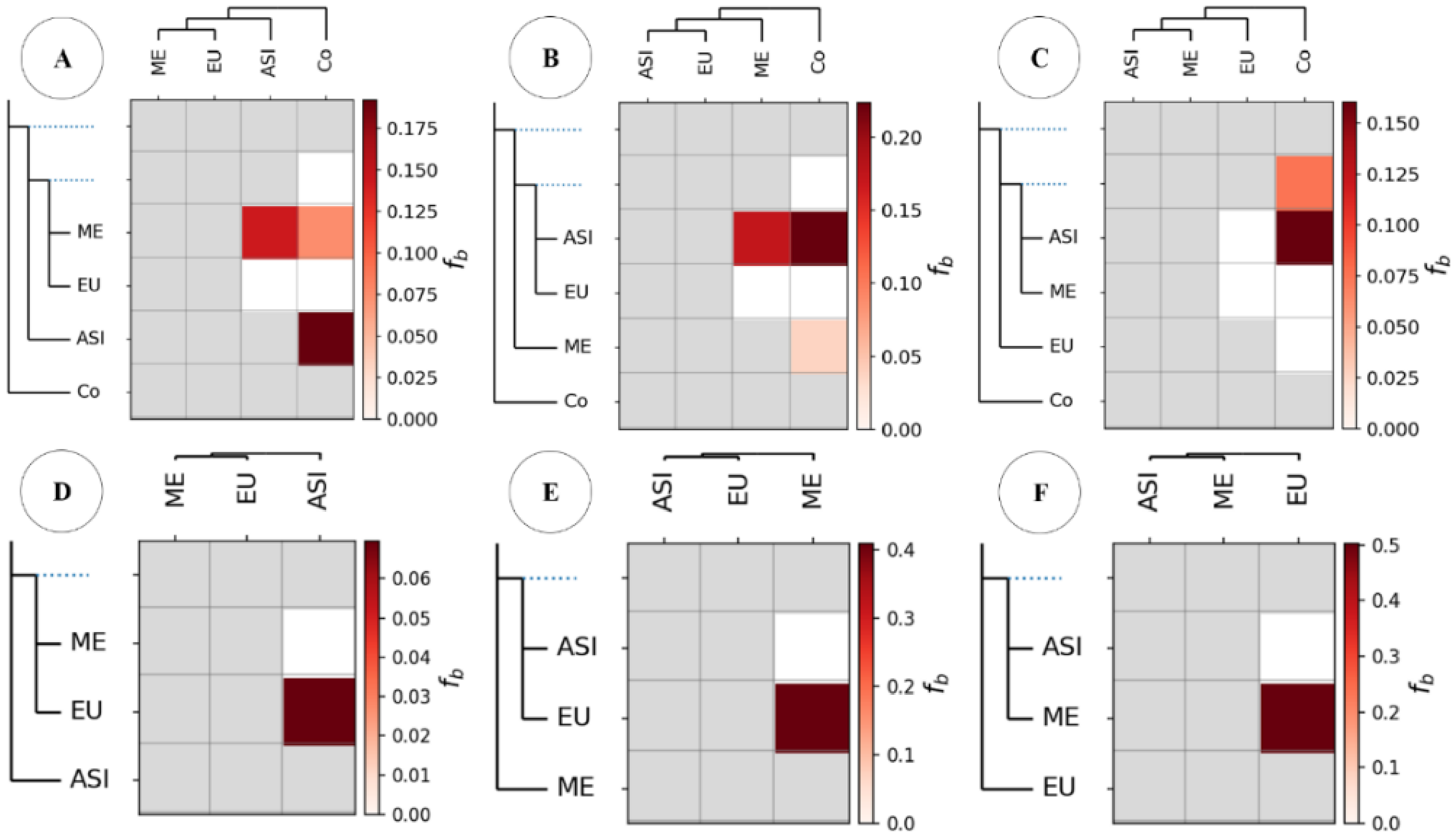

Figure S7. Fbranch matrix plotted using Dsuite f4-statistics results for different tree topologies of parental species and lineages of *C. bursa-pastoris* for subgenome O. a for ((((ME,EU),ASI),Co),Cgr-Outgroup) tree; b for ((((ASI,EU),ME),Co),Cgr-Outgroup) tree; c for ((((ASI,ME),EU),Co),Cgr-Outgroup) tree; d for (((ME,EU),ASI),Co-Outgroup) tree; e for (((ASI,EU),ME),Co-Outgroup) tree; f for (((ASI,ME),EU),Co-Outgroup) tree. Co – C. orientalis, Cgr – *C. rubella/C. grandiflora*, and ASI, ME, EU – lineages of *C. bursa-pastoris*.

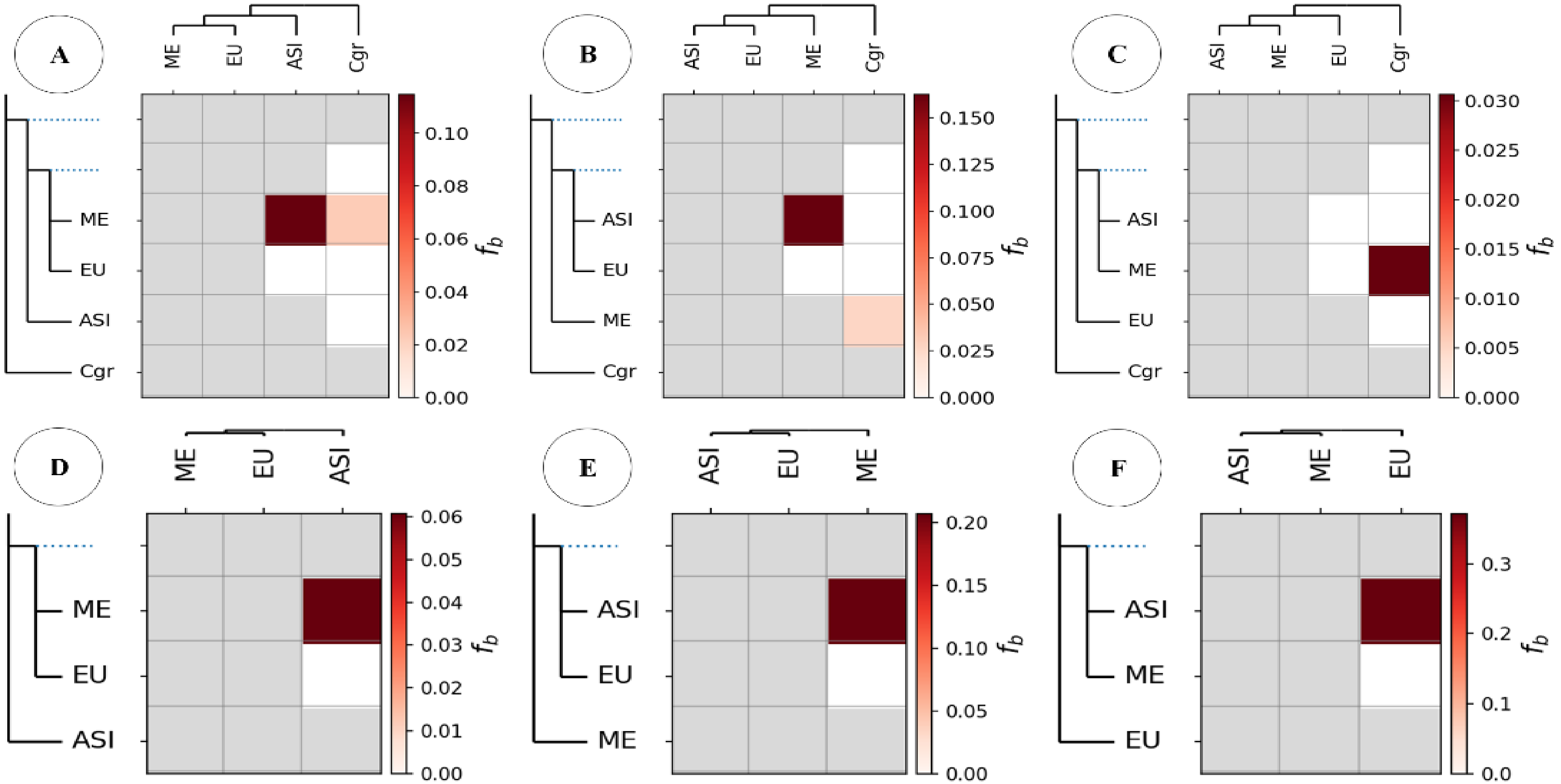

Figure S8. Fbranch matrix plotted using Dsuite f4-stastics results for different tree topologies of parental species and lineages of *C. bursa-pastoris* for subgenome R. a for ((((ME,EU),ASI),Cgr),Co-Outgroup) tree; b for ((((ASI,EU),ME),Cgr),Co-Outgroup) tree; c for ((((ASI,ME),EU),Cgr),Co-Outgroup) tree; d for (((ME,EU),ASI),Cgr-Outgroup) tree; e for (((ASI,EU),ME),Cgr-Outgroup) tree; f for (((ASI,ME),EU),Cgr-Outgroup) tree. Co – *C. orientalis*, Cgr – *C. rubella/C. grandiflora*, and ASI, ME, EU – lineages of *C. bursa-pastoris*.

Mapping Illumina sequencing reads of the lel line to the reference genome using CLC Genomics Workbench 20.0.3

Parameters: Match score = 1, Mismatch cost = 3, Cost of insertions and deletions = Linear gap cost, Insertion cost = 3, Deletion cost = 3, Length fraction = 1.0, Similarity fraction = 0.93, Non-specific match handling = Ignore

Alignment

Total mapped reads ~58-63%, in pairs ~51-58%, >7× average coverage

Basic variant detection 2.1. using CLC Genomics Workbench 20.0.3

Parameters: Ploidy = 2, Ignore positions with coverage above = 100, Ignore broken pairs = Yes, Ignore non-specific matches = Reads, Minimum coverage = 2, Minimum count = 4, Minimum frequency (%) = 15.0, Base quality filter = Yes, Neighborhood radius = 5, Minimum central quality = 20, Minimum neighborhood quality = 15 Read direction filter = Yes, Direction frequency (%) = 5.0, Relative read direction filter = Yes, Significance (%) = 1.0, Read position filter = Yes, Significance (%) = 1.0

Filtration by the SNP database using following rules:

- All polymorphic sites with no matching record in the database were deleted.
- If the variant had been found in the database but not in the F2 plant, its coverage was checked. If the coverage was ≥4, then the reference allele was inserted, else the marker for this plant was set as unidentified.
- Only markers that were present in 90% of the F2 plants were taken.
- Only markers that passed the 1:1 ratio of alleles in F2 according to Pearson’s criterion were taken.
- To view and analyze alleles were coded as “-1” for the reference, “1” for the alternative, “0” for heterozygote, and “-“ for the markers with an unknown state.

Figure S9. F2 data processing.

Mapping Illumina sequencing reads of the lel line to the reference genome using CLC Genomics Workbench 20.0.3

Parameters: Match score = 1, Mismatch cost = 3, Cost of insertions and deletions = Linear gap cost, Insertion cost = 3, Deletion cost = 3, Length fraction = 1.0, Similarity fraction = 0.93, Non-specific match handling = Ignore

↓

Alignment

Total mapped reads 55.30%, in pairs 50.05%, 16× average coverage

↓

Basic variant detection 2.1. using CLC Genomics Workbench 20.0.3

Parameters: Ploidy = 2, Ignore positions with coverage above = 100, Ignore broken pairs = Yes, Ignore non-specific matches = Reads, Minimum coverage = 4, Minimum count = 4, Minimum frequency (%) = 100.0, Base quality filter = Yes, Neighborhood radius = 5, Minimum central quality = 20, Minimum neighborhood quality = 15, Read direction filter = Yes, Direction frequency (%) = 5.0, Relative read direction filter = Yes, Significance (%) = 1.0, Read position filter = Yes, Significance (%) = 1.0

↓

Total variants database

SNV - 254283, MNV - 3370, Insertion - 13252, Deletion – 16984, 12.2× average SNP coverage.

↓

SNP database

Use only SNV - 254283, Frequency ~0.82 on 1kbp

Figure S10. Building the SNP Database.
